# High expression of MMP28 indicates unfavorable prognosis in pancreatic cancer

**DOI:** 10.1097/MD.0000000000025320

**Published:** 2021-03-26

**Authors:** Zhitao Chen, Jiacheng Huang, Mengxia Li, Lele Zhang, Dalong Wan, Shengzhang Lin

**Affiliations:** a Shulan (Hangzhou) Hospital Affiliated to Zhejiang Shuren University, Shulan International Medical College; b Division of Hepatobiliary and Pancreatic Surgery, Department of Surgery, First Affiliated Hospital, School of Medicine; c School of Medicine, Zhejiang University; d Key Laboratory of Combined Multi-organ Transplantation, Hangzhou; e Key Laboratory of the Diagnosis and Treatment of Organ Transplantation, Research Unit of Collaborative Diagnosis and Treatment for Hepatobiliary and Pancreatic Cancer, Chinese Academy of Medical Sciences; f Key Laboratory of Organ Transplantation, Zhejiang Province, Hangzhou, China.

**Keywords:** gene set enrichment analysis, matrix metalloproteinase 28, pancreatic cancer, prognosis

## Abstract

To investigate the expression pattern and diagnostic performance of matrix metalloproteinase 28 (MMP28) in pancreatic cancer (PC).

The RNA-seq data of PC and normal pancreas tissue were acquired from The Cancer Genome Atlas (TCGA) and Genotype-Tissue Expression. Clinical information of PC that included prognostic data was obtained from TCGA. Later, Fisher exact test was applied for comparison of different clinicopathological features between high and low expression of MMP28 in PC. Afterwards, Kaplan-Meier survival analysis and Cox analysis (univariate and multivariate analysis) were used to explore the prognostic performance of MMP28 in PC cohort. Finally, gene set enrichment analysis (GSEA) revealed the potential signaling pathways related to high expression of MMP28 in PC.

Upregulation of MMP28 was identified in PC tissue compared to normal pancreas tissue (*P* < .001). Overexpression of MMP28 was related to histological grade (*P* < .001), M classification (*P* = .014), and survival status (*P* = .028). Kaplan-Meier survival analysis revealed that high level of MMP28 implied unfavorable prognosis in PC (*P* = .002). Multivariate analysis confirmed that MMP28 was an independent risk factor in PC (hazard rate = 1.308, *P* = .018). Our GSEA analysis found that signaling pathways including glycolysis, p53 pathway, notch signaling, estrogen response late, cholesterol homeostasis, estrogen response early, mitotic spindle, and transforming growth factor beta signaling were enriched in the group with higher MMP28 expression.

High expression of MMP28 could be identified in PC, which also served as an independent risk element for PC.

## 1 Introduction

Pancreatic cancer (PC) is a kind of archenteric malignancy with unfavorable short-term and long-term prognosis. According to global cancer statistics in 2018, new cases of PC reached 458,918, and 432,242 cases died from PC.^[1]^ The mortality of PC is the seventh deadliest cause among all cancer, ranking behind lung cancer, colorectum cancer, liver cancer, breast cancer, and esophagus cancer. Traditionally, treatment of surgical operation is the only executable strategy for patients with PC. Approximately 85% PC cases are, however, diagnosed at advanced stage or grade for the scarcity of specific symptom at early stage and therefore are deprived of surgery chances. Imaging methods, such as computer tomography, magnetic resonance cholangiopancreatography based on magnetic resonance imaging, and needle biopsy (fine needle aspiration cytology and needle core biopsy) guided by endoscopic ultrasound, are indispensable for the diagnosis of PC. Although much progress has been made for early diagnosis of PC when applying these imaging modalities, there is still insufficiency in some specific cases.^[2–4]^ Therefore, exploration of biomarkers which exhibits prognosis value for the diagnosis of PC remains unmet.

For the past few decades, much attentiveness has been paid on matrix metalloproteinases (MMPs) for their extensive role in numerous pathological conditions. Twenty-eight members are identified in MMPs family. MMPs, zinc-dependent endopeptidases, which have the ability to degrade components in extracellular matrix (ECM). The activation of MMPs contributes to remodeling of ECM, thus regulating mechanotransduction between ECM and cells and leading to disproportional response to ECM remodeling. Several signaling pathways are involved in such progress, such as phosphatidylinositol 3-kinase pathway, Hippo pathway, integrin signaling pathway, and mitogen-activated protein kinase/extracellular signal-regulated kinase pathway. The aberrant level of MMPs is related to multiple pathological circumstances, such as fibrosis,^[5–7]^ vascular diseases,^[8–10]^ immunity disorder,^[11,12]^ and cancer.^[13,14]^ Matrix metalloproteinase 28 (MMP28), also known as epilysin, is one of the MMPs protein family which locates at 17q11.2.^[15,16]^ It was reported that MMP28 played a role in several pathological processes, like pulmonary fibrosis^[17–20]^ and cancer.^[21,22]^ It has been nearly 20 years since MMP28 was first reported, but the expression pattern and prognostic value of MMP28 was less reported, and the cognition of the function and mechanism of MMP28 in cancer are still limited up to now. The expression pattern and prognostic value of MMP28 in PC remains further probed.

Herein, the expression level of MMP28 mRNA was measured between PC and healthy controls. Secondly, the relationship between the MMP28 expression and multiple clinicopathological characteristics as well as overall survival (OS) was assessed. Thirdly, Kaplan-Meier survival curses were drawn based on different clinicopathological features when log-rank test was applied. Afterwards, univariate and multivariate analysis was performed to probe the prognostic performance of MMP28 in PC. Finally, gene set enrichment analysis (GSEA) was conducted to reveal the potential biological process in patients with PC with higher expression of MMP28.

## 2 Materials and methods

### 2.1 Data acquisition and preparation

TCGAbiolinks package^[23–25]^ in R version 3.6.1 was used to download the clinical information and RNA-seq data of pancreatic adenocarcinoma (PAAD) from The Cancer Genome Atlas (TCGA) (https://portal.gdc.cancer.gov/). In addition, RNA-seq data of healthy pancreatic tissue were also downloaded from Genotype-Tissue Expression (GTEx) (https://www.gtexportal.org/home/). All counts in TCGA and GTEx were transformed to log2(x+1). All Ensemble ID of genes were transformed to gene symbol according to the annotation file “Homo_sapiens.GRCh38.100.chr.gtf” downloaded from Ensembl database (http://asia.ensembl.org/index.html). Guidelines and ethical principles were strictly complied when using TCGA and GTEx data.

### 2.2 Statistical analysis

Pearson chi-square test was applied to assess the relationship between different clinicopathological features and the expression of MMP28, and Fisher exact test was alternative when there was at least 1 expected count <5. Boxplots were generated based on the relative expression value of MMP28. In addition, the area under the curve (AUC) value was computed based on receiver operating characteristic (ROC) analysis when utilizing pROC package^[26]^ in R version 3.6.1. Survival analysis was performed based on survminer package in R version 3.6.1 and log-rank test was applied for the comparison of Kaplan-Meier survival curves between 2 groups. All the visualization was based on ggplot2 package in R version 3.6.1. The “High” and “Low” expression of MMP28 were grouped by the average value of MMP28 in all cancer patients. Univariate analysis and multivariate analysis were performed based on Cox proportional hazard regression. *P* value <.05 was considered statistically significant.

### 2.3 Gene set enrichment analysis

GSEA is an algorithm which figures out if preset gene sets display statistically significant differences between 2 different phenotypes or groups.^[27,28]^ GSEA software version 4.0.3 was downloaded to analyze the RNA-seq expression profile from TCGA cohort. All Ensemble ID of genes were transformed to gene symbol according to the annotation file “Homo_sapiens.GRCh38.100.chr.gtf” download from Ensembl database (http://asia.ensembl.org/index.html). The patients with PC in TCGA cohort were divided into 2 groups (high expression of MMP28 and low expression of MMP28) according to average expression of MMP28. “h.all.v7.1.symbols.gmt” was defined as a priori gene set which clearly records biological process or status. Other parameters in GSEA software were set at default. Nominal (NOM) *P* value <.05 and false discovery rate (FDR) *q* value <0.25 were defined as significant.

### 2.4 Specimens collection and immunohistochemistry

The study was approved by Ethics Committee of the Shulan (Hangzhou) Hospital. Ten PC tissues and their corresponding paracancerous tissues were collected from Shulan (Hangzhou) Hospital. The protocol of immunohistochemistry was described as before.^[29]^ MMP28 (Cat# K008190P, Solarbio) was served as the primary antibody.

## 3 Results

### 3.1 The clinicopathological features of the patients with PC included

Totally 182 samples including 178 tumor samples and 4 paracancerous samples were recorded when PAAD RNA-seq data were downloaded from TCGA. As for clinical information, 185 PC cases were recorded and 8 cases were excluded for the lack of MMP28 expression value. It was showed that the expression condition of MMP28 was associated with histological grade (*P* < .001), M classification (*P* = .014), and survival status (*P* = .028). The relationship between other clinicopathological characteristics and the expression condition of MMP28 are exhibited in Table 1.

**Table 1:** Clinical features of the pancreatic cancer patient in The Cancer Genome Atlas cohort and the relationship between clinical characteristics and expression of matrix metalloproteinase 28.

			MMP28 (%)			
Characteristic	Variable	n (%)	High	Low	χ^2^	*P*
Age	<55 yr	33 (18.64%)	17	16	0.003	.953
	>−55 yr	144 (81.36%)	75	69		
Sex	Male	97 (54.8%)	51	46	0.031	.860
	Female	80 (45.2%)	41	39		
Alcohol consumption history	No	64 (36.16%)	34	30	0.042	.837
	Yes	101 (57.06%)	52	49		
	Not available	12 (6.78%)				
Anatomic location	Head of pancreas	138 (77.97%)	69	69	7.150	.067
	Body of pancreas	14 (7.91%)	10	4		
	Tail of pancreas	14 (7.91%)	10	4		
	Other	11 (6.21%)	3	8		
Histological type	Undifferentiated carcinoma	1 (0.56%)	0	1	5.342^∗^	.106
	Colloid carcinoma	4 (2.26%)	1	3		
	Adenocarcinoma other subtype	25 (14.12%)	9	16		
	Adenocarcinoma ductal type	146 (82.49%)	81	65		
	Discrepancy	1 (0.56%)				
Histological grade	G1	31 (17.51%)	7	24	19.923^∗^	<.001
	G2	94 (53.11%)	50	44		
	G3	48 (27.12%)	34	14		
	G4	2 (1.13%)	0	2		
	Gx	2 (1.13%)	1	1		
Stage	I	21 (11.86%)	8	13	2.837^∗^	.432
	II	146 (82.49%)	78	68		
	III	3 (1.69%)	2	1		
	IV	4 (2.26%)	3	1		
	Not available	3 (1.69%)				
T classification	T1	7 (3.95%)	4	3	2.706^∗^	.660
	T2	24 (13.56%)	10	14		
	T3	141 (79.66%)	76	65		
	T4	3 (1.69%)	2	1		
	TX	1 (0.56%)	0	1		
	Not available	1 (0.56%)				
N classification	N0	49 (27.68%)	27	22	1.291^∗^	.544
	N1	123 (69.49%)	64	59		
	NX	4 (2.26%)	1	3		
	Not available	1 (0.56%)				
M classification	M0	79 (44.63%)	32	47	7.742^∗^	.014
	M1	4 (2.26%)	3	1		
	MX	94 (53.11%)	57	37		
Residual tumor	R0	106 (59.89%)	49	57	2.844^∗^	.418
	R1	52 (29.38%)	31	21		
	R2	5 (2.82%)	3	2		
	RX	4 (2.26%)	2	2		
	Not available	10 (5.65%)				
Survival status	Dead	58 (32.77%)	37	21	4.825	.028
	Alive	119 (67.23%)	55	64		

MMP28 = matrix metalloproteinase 28.

∗ Fisher exact test was applied when there were at least 1 expected count <5.

### 3.2 The expression pattern and ROC analysis of MMP28 in PC

**Figure 1:**
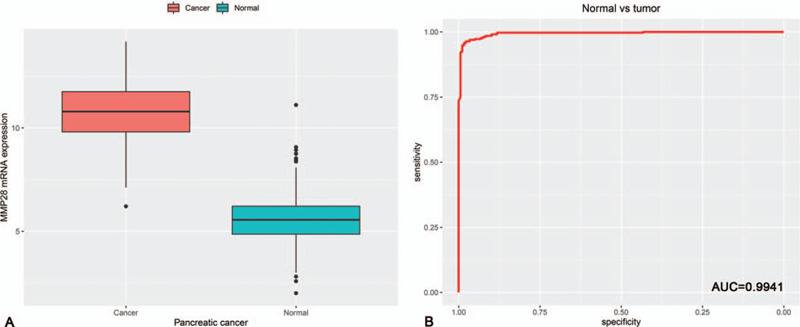
The expression pattern of MMP28 in pancreatic cancer, which was presented by box plot and receiver operating characteristic (ROC) curve. A, Box plot; (B) ROC curve. AUC = area under the curve, MMP28 = matrix metalloproteinase 28.

**Figure 2:**
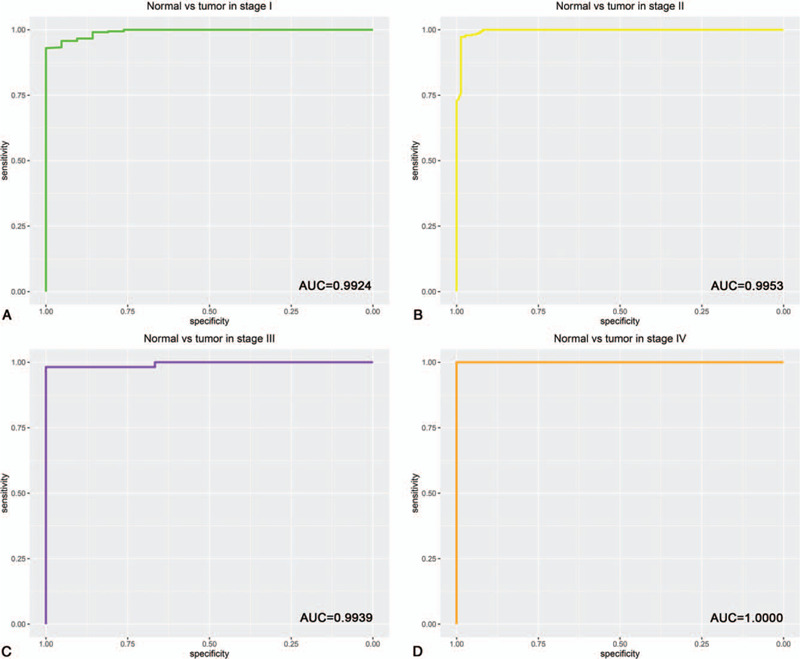
receiver operating characteristic (ROC) analysis of matrix metalloproteinase 28 (MMP28) expression in pancreatic cancer. The expression of MMP28 in normal pancreas tissue was compared to that in stage I (A), stage II (B), stage III (C), and stage IV (D). AUC = area under the curve.

**Figure 3:**
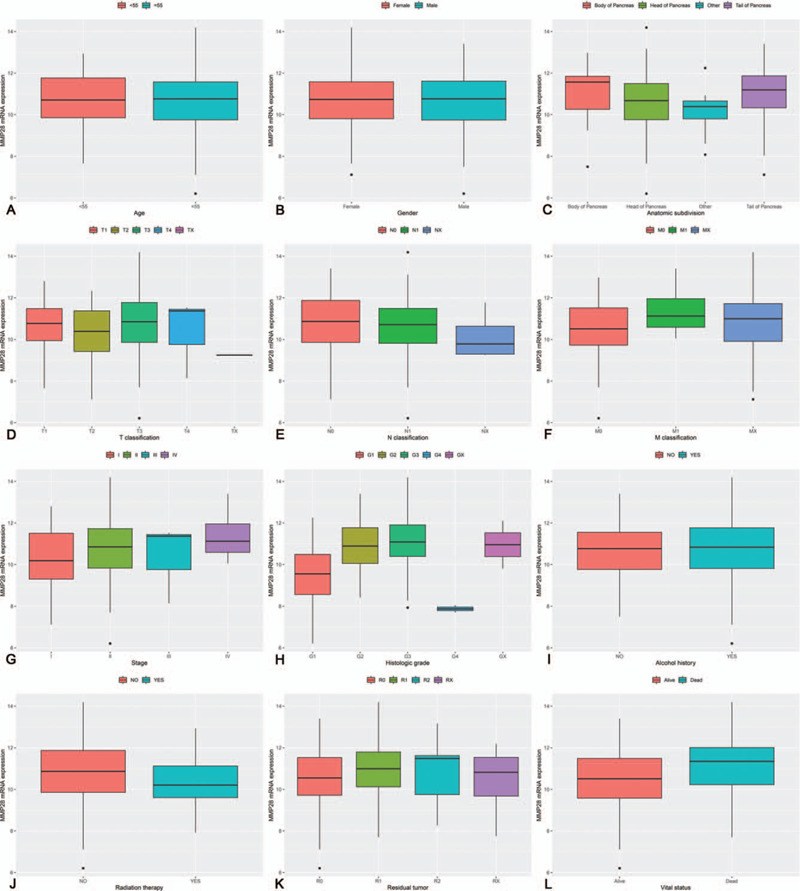
Box plots of matrix metalloproteinase 28 (MMP28) in multiple clinicopathological features. A, Age; (B) sex; (C) anatomic subdivision; (D) T classification; (E) N classification; (F) M classification; (G) stage; (H) histologic grade; (I) alcohol history; (J) radiation therapy; (K) residual tumor; (L) vital status.

A total of 328 normal pancreas samples were downloaded from GTEx database. Box plot and independent samples *T* test suggested that the expression of MMP28 mRNA in PC tissue was significantly higher than that in normal pancreas tissue (*P* < .001) (Fig. 1A). ROC analysis manifested the AUC value of 0.9941 (Fig. 1B). Concretely speaking, the AUC values were 0.9924, 0.9953, 0.9939, and 1.0000 in PC stage I, II, III, and IV, correspondingly (Fig. 2). In addition, the expression of MMP28 in different groups of clinicopathological features is displayed in Figure 3.

### 3.3 Prognostic practice of the overexpression of MMP28 in PC

**Figure 4:**
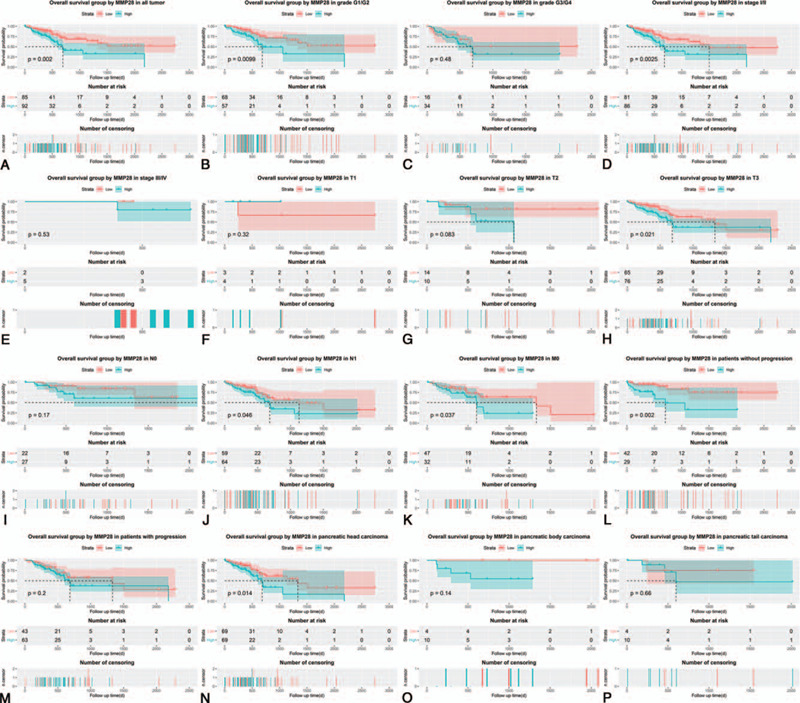
Kaplan-Meier survival curses in pancreatic cancer with different clinical features. A, All tumor; (B) histologic grade G1 and G2; (C) histologic grade G3 and G4; (D) stage I and II; (E) stage III and IV; (F) T1; (G) T2; (H) T3; (I) N0; (J) N1; (K) M0; (L) without progression; (M) with progression; (N) pancreatic head carcinoma; (O) pancreatic body carcinoma; (P) pancreatic tail carcinoma.

First of all, we evaluated the prognostic performance of different expression level of MMP28 in all PC cases, finding the Kaplan-Meier survival curve was separate significantly with *P* value of .002 (Fig. 4A). Afterwards, Kaplan-Meier survival analysis was used in patients with different phenotypes (Fig. 4B–P), for example, histological grade (G1/G2) (*P* = .0099), histological grade (G3/G4) (*P* = .4800), stage I and II (*P* = .0025), stage III and IV (*P* = .5300), T1 (*P* = .3200), T2 (*P* = .0830), T3 (*P* = .0210), N0 (*P* = .1700), N1 (*P* = .0460), M0 (*P* = .0370), without progression (*P* = .0020), with progression (*P* = .2000), pancreatic head carcinoma (*P* = .0140), pancreatic body carcinoma (*P* = .1400), and pancreatic tail carcinoma (*P* = .6600).

In addition, to figure out whether the expression of MMP28 as well as all clinicopathological characteristics served as independent risk elements in patients with PC, univariate analysis and multivariate analysis were employed based on Cox proportional-hazard regression (Table 2). In the part of univariate analysis, age, histologic grade, patients with progression, and the expression level of MMP28 served as risk factors for OS in patients with PC, with the corresponding hazard rate (HR) values of 1.030 (95% confidence interval [CI]: 1.003, 1.057) (*P* = .029), 1.435 (95% CI: 1.064, 1.936) (*P* = .018), 1.994 (95% CI: 1.101, 3.612) (*P* = .023), and 1.487 (95% CI: 1.214, 1.821) (*P* < .001), whereas variables such as histological type (HR: 0.547, *P* = .003), tumor location (HR: 0.660, *P* = .034), and radiation therapy (HR: 0.334, *P* = .007) indicated better prognosis in patients. The variables described above were further included into multivariate analysis. Histological type (HR = 0.640, *P* = .031), tumor location (HR = 0.603, *P* = .015), and radiation therapy (HR = 0.338, *P* = .009) were statistically significant with HR < 1, whereas histologic grade (HR = 1.544, *P* = .033) and the expression value of MMP28 (HR = 1.308, *P* = .018) were independent risk factors in patients with PC.

**Table 2:** Cox analysis of pancreatic cancer in The Cancer Genome Atlas cohort based on univariate and multivariate analysis.

	Univariate analysis		Multivariate analysis	
Parameters	HR (95% CI)	*P*	HR (95% CI)	*P*
Age	1.030 (1.003, 1.057)	.029	1.024 (0.998, 1.051)	.065
Sex (female/male)	1.237 (0.730, 2.096)	.428		
Histological type (ductal type/colloid/undifferentiated/other subtype)	0.547 (0.365, 0.818)	.003	0.640 (0.427, 0.961)	.031
T classification (T1/T2/T3/T4/TX)	1.287 (0.846, 1.959)	.239		
N classification (N0/N1/NX)	1.479 (0.897, 2.439)	.125		
M classification (M0/M1/MX)	0.835 (0.641, 1.088)	.181		
Stage (I/II/III/IV)	1.218 (0.737, 2.013)	.441		
Histologic grade (G1/G2/G3/G4)	1.435 (1.064, 1.936)	.018	1.544 (1.035, 2.301)	.033
Progression (No/Yes)	1.994 (1.101, 3.612)	.023	1.241 (0.647, 2.382)	.516
Alcohol history (No/Yes)	1.678 (0.937, 3.005)	.082		
Tumor location (head of pancreas/body of pancreas/tail of pancreas/other)	0.660 (0.449, 0.969)	.034	0.603 (0.401, 0.908)	.015
Family history of cancer (no/yes)	1.339 (0.677, 2.648)	.402		
History of chronic pancreatitis (no/yes)	1.120 (0.478, 2.624)	.794		
History of diabetes (no/yes)	0.843 (0.434, 1.635)	.613		
Radiation therapy (no/yes)	0.334 (0.151, 0.739)	.007	0.338 (0.149, 0.766)	.009
Residual tumor (R0/R1/R2/RX)	1.267 (0.879, 1.826)	.205		
MMP28	1.487 (1.214, 1.821)	<.001	1.308 (1.048, 1.633)	.018

HR = hazard rate, MMP28 = matrix metalloproteinase 28.

### 3.4 Biological process related to high MMP28 expression based on GSEA

**Figure 5:**
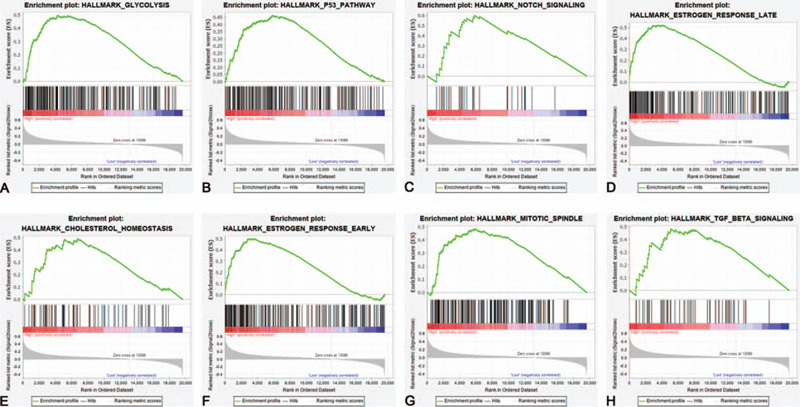
Gene Set Enrichment Analysis (GSEA) of high expression of matrix metalloproteinase 28 (MMP28). A, Glycolysis; (B) p53 pathway; (C) notch signaling; (D) estrogen response late; (E) cholesterol homeostasis; (F) estrogen response early; (G) mitotic spindle; (H) transforming growth factor beta (TGF-β) signaling.

There were 50 gene sets preset in “h.all.v7.1.symbols.gmt.” In the phenotype group of overexpression of MMP28, 47 gene sets were upregulated. Fifteen gene sets are significant at FDR < 0.25. Four gene sets are significantly enriched at NOM *P* value <0.01, and 8 gene sets are significantly enriched at NOM *P* value <0.05. As shown in Table 3, the most significant signaling pathways which met the criteria (NOM *P* value <.05 and FDR *q* value <0.25) were glycolysis, p53 pathway, notch signaling, estrogen response late, cholesterol homeostasis, estrogen response early, mitotic spindle, and transforming growth factor beta (TGF-β) signaling (Fig. 5).

**Table 3:** Biological process related to high expression of matrix metalloproteinase 28 in pancreatic cancer based on GSEA analysis.

NAME	SIZE	ES	NES	NOM *P*	FDR *q*
HALLMARK_GLYCOLYSIS	200	0.4939	1.9712	.0020	0.0473
HALLMARK_P53_PATHWAY	199	0.4630	1.8634	.0060	0.0814
HALLMARK_NOTCH_SIGNALING	32	0.5989	1.8535	.0020	0.0575
HALLMARK_ESTROGEN_RESPONSE_LATE	200	0.5204	1.7891	.0019	0.0827
HALLMARK_CHOLESTEROL_HOMEOSTASIS	74	0.4905	1.7790	.0185	0.0685
HALLMARK_ESTROGEN_RESPONSE_EARLY	200	0.5000	1.7182	.0101	0.0861
HALLMARK_MITOTIC_SPINDLE	199	0.4822	1.6841	.0182	0.0802
HALLMARK_TGF_BETA_SIGNALING	54	0.4839	1.6481	.0329	0.0917

ES = enrichment score, FDR = false discovery rate, GSEA = Gene Set Enrichment Analysis, NES = normalized enrichment score, NOM = nominal.

### 3.5 Immunohistochemistry staining validation

**Figure 6:**
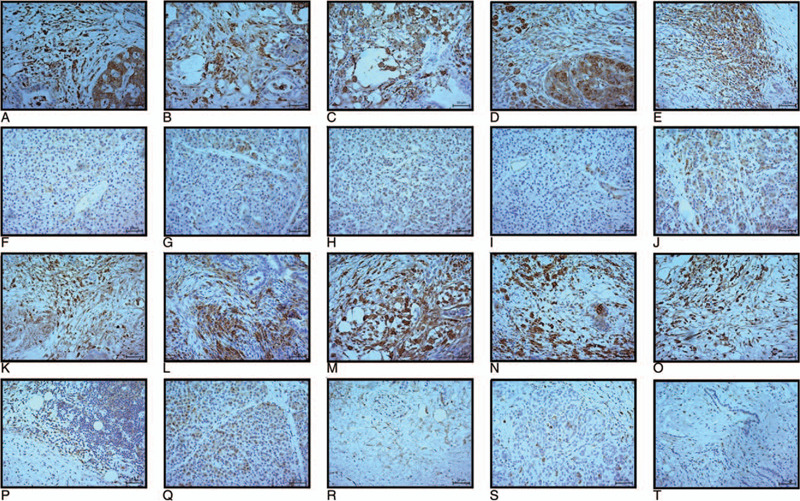
Matrix metalloproteinase 28 (MMP28) immunohistochemistry image of pancreatic tissue and the corresponding paracancerous tissue. A–E and K–O, Pancreatic tissue. F–J and P–T, Corresponding paracancerous tissue.

Our immunohistochemistry staining validated that MMP28 was upregulated in PC compared to corresponding paracancerous tissue. The image is displayed in Figure 6.

## 4 Discussion

Our present work was to assess the expression pattern and prognostic performance of MMP28 in PC. High expression of MMP28 was identified based on the cohort with 178 PC tissue and 332 normal pancreas tissue. ROC analysis based on MMP28 displayed high diagnostic consistency and accuracy. Overexpression of MMP28 was correlated with histological grade, M classification, and survival status. As for prognostic performance, MMP28, as well as histologic grade, served as independent hazard indicators for patients with PC.

MMPs family proteins, which have the ability to degrade almost all ECM components, perform vital functions in multiple types of cancer. Gobin et al^[30]^ performed a comprehensive study of the diagnostic or prognostic value of MMPs based on TCGA cohort, finding the aberrant expression and prognostic value of MMPs in multiple types of cancer. The dysregulated expression of MMPs was reported in breast cancer,^[31]^ hepatocellular carcinoma,^[32]^ PC,^[33]^ and so on. In addition, several studies reported the prognostic value of MMPs in cancer, such as MMP11 in PC,^[34]^ MMP2 in hepatocellular carcinoma,^[34]^ MMP1 in cervical squamous cell carcinoma,^[35]^ and so on. From the perspective of mechanism research, more information of MMPs in cancer was unveiled. The activity of MMPs could be regulated by angiogenesis-associated growth factors which were secreted by tumor cells, leading to angiogenic chaos in tumor microenvironment.^[36]^ In addition, the hypoxia in tumor microenvironment regulated the synthesis of MMPs, contributing to epithelial-mesenchymal transition (EMT) and cell proliferation.^[37]^ Degradation of ECM proteins also increased the probability of the metastasis of cancer cells.^[38]^

PC is characterized as malignancy accompanying matrix stiffness, and a large number of researches concluded that PC tissue was stiffer than healthy pancreas tissue.^[39–41]^ The complexity of the relationship between PC and MMPs is worth discussing. Dysregulation of MMPs in PC frequently indicated poor prognosis.^[33,42,43]^ Downregulation of feline sarcoma-related/signal transducer and activator of transcription 3/MMP2 pathway suppressed the metastasis and invasion of PC.^[44]^ Protein kinase D2 stimulated MMP7 and MMP9, leading to the invasion of pancreatic cells.^[45]^

Although much attention was paid to MMPs, it remained elusive what role MMP28 might play in cancer. The transcription of MMP28 was suppressed by Krüppel-like factor 9, which therefore inhibited the metastasis and invasion of gastric cancer cells.^[22]^ In hepatocellular carcinoma, upregulation of MMP28 was associated with unfavorable prognosis and Notch3 signaling was indispensable when MMP28 exerted its function.^[21]^ MMP28 was, however, rarely studied in PC. High expression of MMP28 was regulated by Rac GTPase activating protein 1 and indicated worse prognosis in PC.^[46]^ Our research supplemented the research topic related to the prognostic performance of MMP28 in PC and further validated the conclusion that high expression of MMP28 was associated with unfavorable prognosis in PC. More studies, especially high-quality meta-analysis, were, however, needed to support this verdict.

Perhaps it intrigues us a lot why upregulation of MMP28 could serve as an independent risk factor in PC. The relationship between MMP28 and primary biological process might partially explain. Our GSEA analysis found that signaling pathways including glycolysis, p53 pathway, notch signaling, estrogen response late, cholesterol homeostasis, estrogen response early, mitotic spindle, and TGF-β signaling were enriched in the group with higher MMP28 expression. In another word, MMP28 was probably involved in the remodeling of ECM, energy metabolism, and cell proliferation. In addition, MMP28 enhanced EMT when TGF-β signaling pathway was activated, and downstream Snail transcription was upregulated.^[47]^ Researchers also reported that the reason why high MMP28 indicated worse prognosis was associated with Notch3 signaling pathway.^[21]^ The mechanism of MMP28 in PC, however, needs to be further elucidated.

## Author contributions

**Conceptualization:** ShengZhang Lin.

**Formal analysis:** Zhitao Chen, Jiacheng Huang.

**Investigation:** Zhitao Chen, Jiacheng Huang, Mengxia Li, Lele Zhang, ShengZhang Lin.

**Methodology:** Zhitao Chen, Jiacheng Huang, ShengZhang Lin.

**Software:** Zhitao Chen, Jiacheng Huang, Mengxia Li, Dalong Wan.

**Supervision:** ShengZhang Lin.

**Visualization:** Jiacheng Huang, Mengxia Li, Lele Zhang, Dalong Wan, ShengZhang Lin.

**Writing – original draft:** Zhitao Chen, Jiacheng Huang.

**Writing – review & editing:** Zhitao Chen, Jiacheng Huang, Mengxia Li, Lele Zhang, Dalong Wan, ShengZhang Lin.
